# Synthesis and Characterization of Carbon and Carbon-Nitrogen Doped Black TiO_2_ Nanomaterials and Their Application in Sonophotocatalytic Remediation of Treated Agro-Industrial Wastewater

**DOI:** 10.3390/ma14206175

**Published:** 2021-10-18

**Authors:** Saifur Rahman, Rab Nawaz, Javed Akbar Khan, Habib Ullah, Muhammad Irfan, Adam Glowacz, Katarzyna Lyp-Wronska, Lukasz Wzorek, Mohammad Kamal Asif Khan, Mohammed Jalalah, Mabkhoot A. Alsaiari, Abdulkarem H. Almawgani

**Affiliations:** 1 Electrical Engineering Department, College of Engineering, Najran University Saudi Arabia, Najran 61441, Saudi Arabia; srrahman@nu.edu.sa (S.R.); miditta@nu.edu.sa (M.I.); msjalalah@nu.edu.sa (M.J.); ahalmawgani@nu.edu.sa (A.H.A.); 2 Fundamental and Applied Sciences (FASD), Universiti Teknologi PETRONAS (UTP), Seri Iskandar 32610, Malaysia; habib_g02832@utp.edu.my; 3 Centre of Innovative Nanostructures and Nanodevices (COINN), Institute of Autonomous System, Universiti Teknologi PETRONAS (UTP), Seri Iskandar 32610, Malaysia; 4 Mechanical Engineering Department, Universiti Teknologi Petronas, Seri Iskandar 32610, Malaysia; 5 Department of Automatic Control and Robotics, Faculty of Electrical Engineering, Automatics, Computer Science and Biomedical Engineering, AGH University of Science and Technology, al. A. Mickiewicza 30, 30-059 Kraków, Poland; adglow@agh.edu.pl; 6 Department of Materials Science and Non-Ferrous Metal Engineering, Faculty of Non-Ferrous Metals, AGH University of Science and Technology, al. Mickiewicza 30, 30-059 Kraków, Poland; klyp@agh.edu.pl; 7 Wzorek.Systems, ul. Kapelanka 10/18, 30-347 Kraków, Poland; lukasz@wzorek.systems; 8 Mechanical Engineering Department, College of Engineering, Najran University Saudi Arabia, Najran 11001, Saudi Arabia; mkkhan@nu.edu.sa; 9 Empty Qaurter Research Unit, Chemistry Department, College of Science and Art at Sharurah, Najran University Saudi Arabia, Najran 61441, Saudi Arabia; mabkhoot.alsaiari@gmail.com

**Keywords:** carbon-nitrogen doped TiO_2_, bandgap, visible light absorption, agro-industrial wastewater, remediation, sonophotocatalysis, chemical oxygen demand, electrical energy consumption

## Abstract

The conventional open ponding system employed for palm oil mill agro-effluent (POME) treatment fails to lower the levels of organic pollutants to the mandatory standard discharge limits. In this work, carbon doped black TiO_2_ (CB-TiO_2_) and carbon-nitrogen co-doped black TiO_2_ (CNB-TiO_2_) were synthesized via glycerol assisted sol-gel techniques and employed for the remediation of treated palm oil mill effluent (TPOME). Both the samples were anatase phase, with a crystallite size of 11.09–22.18 nm, lower bandgap of 2.06–2.63 eV, superior visible light absorption ability, and a high surface area of 239.99–347.26 m^2^/g. The performance of CNB-TiO_2_ was higher (51.48%) compared to only (45.72%) CB-TiO_2_. Thus, the CNB-TiO_2_ is employed in sonophotocatalytic reactions. Sonophotocatalytic process based on CNB-TiO_2_, assisted by hydrogen peroxide (H_2_O_2_), and operated at an ultrasonication (US) frequency of 30 kHz and 40 W power under visible light irradiation proved to be the most efficient for chemical oxygen demand (COD) removal. More than 90% of COD was removed within 60 min of sonophotocatalytic reaction, producing the effluent with the COD concentration well below the stipulated permissible limit of 50 mg/L. The electrical energy required per order of magnitude was estimated to be only 177.59 kWh/m^3^, indicating extreme viability of the proposed process for the remediation of TPOME.

## 1. Introduction

Palm oil production is a multistage process that generates an enormous amount of wastewater, commonly termed as palm oil mill effluent (POME). According to an estimate, the annual production of POME in Malaysia is approximately 48–72 million metric tons [1]. To maintain a clean and pleasant environment, the palm oil industries are obliged to treat POME to comply with the stipulated discharge limits of 20 mg/L and 50 mg/L for biological oxygen demand (BOD) and chemical oxygen demand (COD), respectively, set by the Department of Environment (DOE) Malaysia [2]. Normally, palm oil industries employ biological treatment (open ponding system) consisting of aerobic and anaerobic biological processes for POME treatment. While these traditional methods can decrease the COD and BOD levels to some extent, the final discharge quality frequently fails to comply with the regulatory discharge limit [3,4]. The presence of harmful organic fractions in TPOME might cause irreversible damage to aquatic ecosystem and the chemical pollution, due to the emission of TPOME contaminated with inorganic and organic pollutants may endanger the aquatic biodiversity [5,6].

Other methods investigated for POME treatment include adsorption, coagulation-flocculation, electrocoagulation, membrane separation, and skimming. These technologies can remove almost 90% of the organic fractions from raw POME [7,8,9,10]. However, they are not likely to replace the most widely employed open ponding system for various reasons, such as high chemical and energy inputs, high treatment and capital cost, long treatment time, and the necessity of skilled labor [11]. The only suitable option is to introduce some form of advanced processes at the remediation stage, to reduce the pollutant level to environmentally acceptable standards. 

Reports indicate a huge potential of photocatalysis for the remediation of TPOME, and are well-proven in previous works [12,13,14,15,16,17,18,19,20]. For instance, Alhaji et al. [12] reported that the immobilized TiO_2_ based photocatalytic process was able to efficiently remove more than 90% of COD from TPOME in 5 h of ultraviolet (UV) irradiation. Another study on photo-mineralization of TPOME using TiO_2_ based photocatalysis demonstrated 78% COD removal from TPOME in 20 h under UV irradiation [21]. Nanostructured transition metal compounds, such as TiO_2_, with high surface-to-volume ratios are of particular scientific interest in the field of photocatalysis for environmental remediation. Furthermore, photocatalysis in combination with other advanced oxidation processes (AOPs) is anticipated to achieve much better results in terms of organic compounds, especially COD removal from TPOME [22].

Photocatalysis assisted by ultrasonication (US) is a more consolidated approach to improve the degradation of pollutants, as cavitation can augment the performance of photocatalysis in two ways: (1) it promotes mass transfer of pollutant molecules from solution to the surface of the photocatalysts, and (2) it produces additional radicals, which are the predominant species taking part in photodegradation [14,23]. In addition, the US helps continuously regenerate the photocatalyst surface and expose active sites for organics adsorption and consequent higher photodegradation. For instance, Parthasarathy et al. [24] studied the effectiveness of combining US cavitation and adsorption for remediation of TPOME. It was reported that combining the two processes resulted in 100% of COD and 84.92% of BOD removal from TPOME. However, adsorption only transfers pollutants from one medium to another without complete degradation. Therefore, it is desirable to combine US with photocatalysis, which could help mineralize organics in TPOME to environmentally benign products, such as carbon dioxide and water.

In most of the previous studies, UV irradiation has been used to activate the TiO_2_ nanomaterials. The degradation efficiency of the TiO_2_ was higher under UV irradiation compared to visible light. However, UV represents only a small portion of the natural sunlight. Efforts have been directed to shift the photo-response of TiO_2_ into the visible region of the sunlight, which accounts for 45% of the radiation. However, the remediation efficiency under visible light is as low as 26.77%. Increasing the visible light performance of TiO_2_ is crucial in order to benefit from the abundantly available solar energy, and make the remediation process sustainable in the long run.

Unlike the previous studies, the present study aims to synthesize carbon doped and carbon-nitrogen co-doped black TiO_2_ nanomaterials which can be activated by visible light, and investigate their performance in the advanced application of photocatalytic and sonophotocatalytic remediation of TPOME. To the best of the authors’ knowledge, it appears that the synthesis of carbon and nitrogen co-doped black TiO_2_ nanomaterials and their application in sonophotocatalytic remediation of TPOME is rarely explored. This study also attempts to increase the remediation of efficiency of TPOME by combining photocatalysis and US cavitation. It appears that such a combination of AOPs was investigated for the first time for the remediation of TPOME. Furthermore, the effect of different experimental conditions, such as initial concentration of COD, TiO_2_ loading, H_2_O_2_ dosage, US frequency, and US power, on COD removal from TPOME was also evaluated. Kinetics studies and reusability tests were also carried out to determine the order of the reactions and stability of the TiO_2_ photocatalyst, respectively.

## 2. Materials and Methods

### 2.1. Materials

Titanium (IV) chlorides (TiCl_4_, 99.9%), urea (CH_4_N_2_O, 99%), glycerol (C_3_H_8_O_3_, 85%), and aqueous ammonia (NH_4_OH, 25%) were purchased from Merck (Darmstadt, Germany). TPOME sample was strategically collected from the release point of a final stabilization pond of the palm oil industry situated in Perak, Malaysia. It was centrifuged at 8000 rpm for 15 min to remove the suspended solids before treatment. The pH and COD concentrations of the TPOME were 7.6 and 800 mg/L, respectively.

### 2.2. Synthesis of Carbon Doped Black TiO_2_

**Figure 1:**
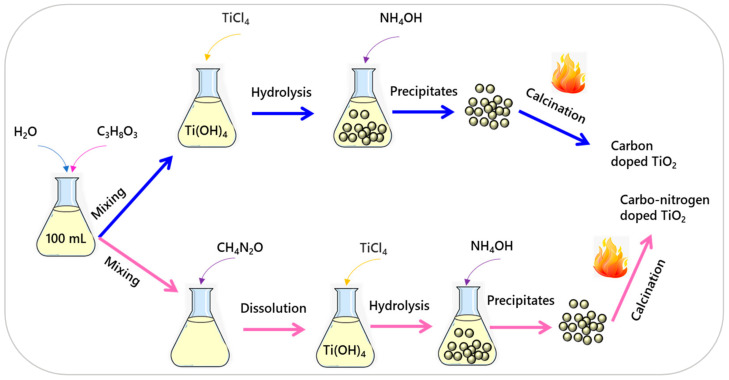
Schematic illustration of the synthesis of CB-TiO_2_ and CNB-TiO_2_ nanoparticles.

Carbon doped black TiO_2_ nanomaterial was synthesized via a modified chemical precipitation method, as reported in previous work [25]. Figure 1 shows a schematic illustration of the synthesis carbon doped black TiO_2_ nanomaterial. TiCl_4_ (Merck, 99.9%) was used as a precursor and 0.10 mole of it were added dropwise into a mixture of water and glycerol (9:1, *v*/*v*) (100 mL) under vigorous stirring. The TiO_2_ precipitates were obtained by adding NH_4_OH (2.5 M) into the Ti(OH)_4_ sol.

The precipitates were recovered by centrifugation at 6000 rpm for 10 min, and washed thoroughly with deionized water to remove residual chloride ions, followed by drying at 80 °C for 24 h in an oven (Memmert, Schwabach, Germany). The dried powder was grinded and calcined at 300 °C for an hour in a muffle furnace (Nabertherm) to get carbon doped black TiO_2_ nanomaterial. For ease of referencing, the carbon doped black TiO_2_ nanomaterial was labeled as CB-TiO_2_.

### 2.3. Synthesis of Carbon and Nitrogen Co-Doped TiO_2_

The carbon and nitrogen doped black TiO_2_ nanomaterial was synthesized via a modified sol-gel technique [26]. A schematic of the synthesis procedure for carbon and nitrogen doped black TiO_2_ nanoparticles is presented in Figure 1. Glycerol and water were mixed in a fixed ratio of 9:1 (*v*/*v*). A specific amount of urea (30.03 g, 499.86 mmol) was dissolved in a water/glycerol mixture. Then, 0.1 moles of TiCl_4_ were slowly added dropwise to the mixture under constant stirring to perform hydrolysis. Afterward, 300 mL of 2.5 M NH_4_OH was added dropwise to the solution to raise to pH 10 to get maximum precipitation. The sol was aged in air for 24 h under continuous stirring, to allow further hydrolysis. Subsequently, the sol was dehydrated at 80 °C for 24 h in an oven and then calcined at 300 °C for 1 h with a heating rate of 5 °C min^−1^. The carbon and nitrogen co-doped black TiO_2_ nanomaterial was labeled as CNB-TiO_2_.

### 2.4. Nanomaterials Characterization

For structural properties determination, the X-ray diffraction (XRD) patterns were recorded using a powder X-ray diffraction spectrometer (Rigaku D/max-RA, Rigaku, Austin, TX, USA). The XRD patterns were obtained using CuKα radiation (λ = 1.5406 Å) at 40 kV and 40 mA in a scanning range of 20‒80° (2θ) with a step size of 0.027°. High-resolution transmission electron microscopy (HRTEM) micrographs were captured using transmission electron microscope (Tecnai G2-F20 X-Twin TMP, FEI, Dawson Creek Drive, Hillsboro, OR, USA) with accelerating voltage of 100 kV. X-ray photoelectron spectroscopy (XPS) analyses were carried out via ESCALAB 250Xi X-ray photoelectron spectrometer. X-rays (Al Kα) were used for the excitation. C1s correction at 284.6 eV was used for calibrating the instrument. The diffuse reflectance UV-Visible (DRUV-Vis) spectra were obtained with UV-Vis spectrometer (Agilent Carry100, Santa Clara, CA, USA), using BaSO_4_ as the reference substance. Photoluminescence (PL) spectra of the samples were recorded using PerkinElmer LS–55 fluorescence spectrophotometer (PerkinElmer, Inc, Waltham, MA, USA). The physio-sorption isotherms of the samples were acquired at ‒196 °C using Micromeritics ASAP 2020 analyzer (Micromeritics®, Norcross, GA, USA). The specific surface area (SSA) of the samples was computed based on Brunauer, Emmett, and Teller’s (BET) method from the adsorption data. The Barret-Joyner-Halenda (BJH) technique was used for the estimation of the pore size.

### 2.5. Experimental Setup

**Figure 2:**
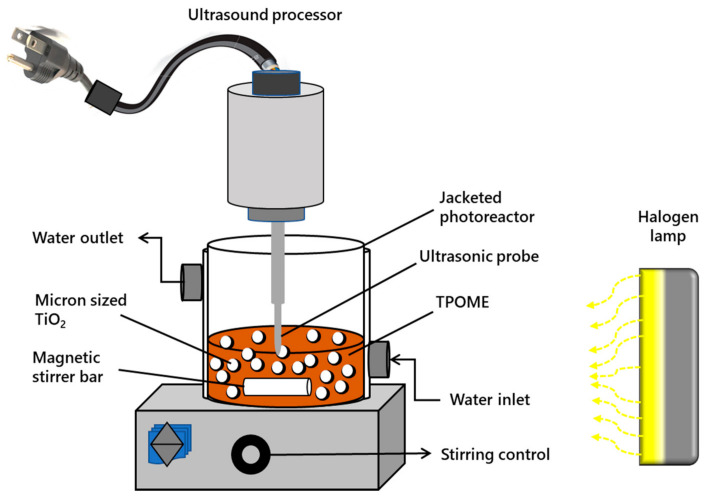
Experimental setup for carrying out TPOME remediation tests.

Laboratory scale experiments in the batch mode were conducted for a combination of different processes (sonolysis, photolysis, sonophotolysis, sonocatalysis, photocatalysis, and sonophotocatalysis). Figure 2 shows the schematic of the experimental setup used for carrying out the reactions. All the experiments were carried out in a 250 mL jacketed cylindrical photoreactor. Water was circulated in a cylindrical photoreactor equipped with a jacket for circulating water to sustain the reaction vessel’s temperature at around 25–30 °C. A halogen lamp with a power of 500 W (λ = 400–650 nm) was used as a visible light source, which was positioned horizontally at a 25 cm distance from the photoreactor.

For determining the best photocatalyst, the performance of the CB-TiO_2_ and CNB-TiO_2_ was evaluated for COD removal from TPOME. The preliminary results confirmed the superior performance of the CNB-TiO_2_. For sonocatalysis, photocatalysis, and sonophotocatalysis, a specific amount of CNB-TiO_2_ (0.4 g/L) was dispersed in 100 mL of TPOME solution (initial COD: 800 mg/L). For the US-assisted reaction, a 30 kHz ultrasonic processor (UP100H, Hielscher, Germany) was placed above the jacketed cylindrical photoreactor and the US probe was immersed in TPOME solution, avoiding contact with the walls of the reaction vessel. A standard sonotrode tip with a diameter of 3 mm and an acoustic power density of 460 Wcm^−2^ was used for generating US cavitation. A pulse sonication (5 pulses separated by 5 s intervals) was maintained throughout the US-assisted experiments.

### 2.6. Experimental Procedures

First, all the reactions were carried out under the same experimental condition of 0.4 g/L photocatalyst loading, 120 min of visible light irradiation, 800 mg/L of initial COD concentration, and pH 7.5 to identify the best photocatalyst. The TPOME samples were then treated using different processes and a combination of different AOPs, including sonolysis, photolysis, sonophotolysis, sonocatalysis, photocatalysis, and sonophotocatalysis. Based on the preliminary results, the best combination of processes (US-assisted photocatalysis or sonophotocatalysis) in terms of COD removal was selected for further investigation.

Then, the effect of various operational variables such as initial COD concentration (varied through dilution; 800, 600, 400, and 200 mg/L), CNB-TiO_2_ loading (0.3, 0.6, 1.2, and 2.4 g/L), and H_2_O_2_ dosage (1.0, 2.0, 5.0, and 10.0 mM) on COD removal from TPOME was investigated. The samples (2.0 mL) were taken from the photoreactor after each run at a specific time interval of 15–180 min. The samples were diluted with DI water (1:5) where needed and filtered via a nylon syringe filter (0.22 μm) to remove any of the residual solid particles before COD measurement. The COD of each withdrawn sample was measured using a spectrophotometer (DR5000, Hach, Loveland, CO, USA) according to the standard method as described in a previous study [27]. The COD removal efficiency was calculated according to Equation (1):

(1) $$\mathrm{Removal}\;\left(\%\right)=\frac{C_{o}-C_{t}}{C_{o}}\times 100\%$$

where C*_o_* is COD concentration before treatment, and C*_t_* is the concentration of COD at a specific sampling interval after the treatment.

In the present study, H_2_O_2_ was used as an oxidant which can interfere and lead to the overestimation of COD measurements [28]. Therefore, H_2_O_2_ was quenched by heating the samples at around 40 °C for a few min to avoid its interference with COD measurement.

### 2.7. Synergy Index

The synergy index (SI) for sonophotolysis and sonophotocatalysis was calculated using Equations (2)–(5), respectively. SI can be defined as the ratio between the combined effect and individual effect of the processes. An SI equal to 1 means no interaction between the two processes; SI > 1 means an increasing positive interaction of the two processes; and SI < 1 shows an increasing negative interaction of the two combined processes.

(2) $$\mathrm{SI}=\frac{k\;\left(sonophotolysis\right)}{k\;\left(sonolysis\right)\;+\;k\;\left(photolysis\right)}$$

(3) $$\mathrm{SI}=\frac{k\;\left(sonocatalysis\right)}{k\;\left(sonolysis\right)\;+\;k\;\left(catalysis\right)}$$

(4) $$\mathrm{SI}=\frac{k\;\left(photocatalysis\right)}{k\;\left(photolysis\right)\;+\;k\;\left(catalysis\right)}$$

(5) $$\mathrm{SI}=\frac{k\;\left(sonophotocatalysis\right)}{k\;(sonolysis\;+\;k\;\left(photocatalysis\right)}$$

where *k* is the rate constant for the respective process.

### 2.8. Recyclability of CNB-TiO_2_

The recoverability of the CNB-TiO_2_ nanomaterial was evaluated. For this purpose, CNB-TiO_2_ nanomaterial was employed in five successive cycles in the sonophotocatalytic process, and the recyclability of the photocatalyst was determined based on percentage removal of COD from TPOME under visible light irradiation. The spent photocatalyst was recovered after each experiment through centrifugation, washed several times with deionized water, and then dried at 80 °C for 24 h before using it in the next run. The same procedures were repeated for five cycles in a row and the recyclability of the CNB-TiO_2_ photocatalyst was determined.

## 3. Results and Discussion

### 3.1. Properties of the CB-TiO_2_ and CNB-TiO_2_ Nanomaterials

**Figure 3:**
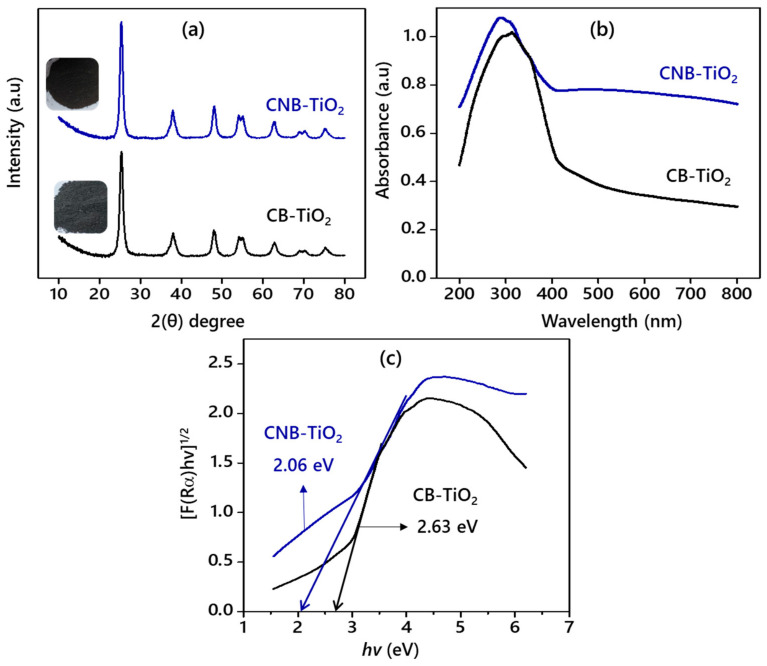
(**a**) XRD patterns, (**b**) UV-vis absorption spectra, and (**c**) T-plot of the bandgap energies of the CB-TiO_2_ and CNB-TiO_2_. Inset of (**a**) photographs of the CB-TiO_2_ and CNB-TiO_2_.

Figure 3a displays the XRD patterns of the CB-TiO_2_ and CNB-TiO_2_. Both of the samples exhibited diffraction peaks typical of anatase crystalline structure (JCPDS 21-1272). The XRD patterns revealed that either carbon or simultaneous carbon and nitrogen doping has no effect on the crystalline structure of TiO_2_ nanomaterials. A closer inspection of Table 1 revealed that the anatase peak of (101) plane was shifted to a lower angle 25.2754° (2θ) in CNB-TiO_2_, along with a slight increase in the d-spacing and full width at half maximum. The CNB-TiO_2_ showed increased d-spacing (0.352 nm) compared to 0.351 nm of CB-TiO_2_. The increase in the d-spacing could be due to the replacement of the oxygen atom by N atom with a large radius [29]. The average crystallite size of CB-TiO_2_ was larger (22.18 nm) than the CNB-TiO_2_ (11.09 nm).

Figure 3b depicts the DRUV-Vis absorption spectra of the CB-TiO_2_ and CNB-TiO_2_. The CNB-TiO_2_ absorbs light across the entire UV and visible light range. The CB-TiO_2_ has a less intense visible light absorption compared to CNB-TiO_2_. Obviously, the enhanced visible light absorption by the CNB-TiO_2_ and the redshift or the extension of the light absorption edge (Table 1) is due to the synergistic effect of simultaneous C and N doping. The light absorption by the samples is consistent with their colors, as presented in the inset of Figure 3a. Regardless of the synthesis method for CB-TiO_2_ and CNB-TiO_2_, the DRUV-Vis results demonstrate that the light absorption is enhanced in both types of TiO_2_ nanomaterials fabricated via organic solvent (glycerol) assisted synthesis methods [30]. However, the light absorption by CNB-TiO_2_ was more pronounced compared to CB-TiO_2_, which could be due to the dark black color of the sample, enabling it to absorb the light across the whole visible and UV light spectrum [31].

The C and N form localized electronic states in the bandgap of CNB-TiO_2_. The localized energy states result in the reduction of the bandgap, consequently leading to a redshift of the absorption edge [32]. A significant reduction in the bandgap to 2.06 eV of the CNB-TiO_2_ from 2.63 eV for CB-TiO_2_ is evident from Figure 3c. This implies that electrons might be easily evacuated from both the impurity state of C and N into the CB of TiO_2_ and from the VB of TiO_2_ into the impurity state of C and N under visible light [33,34].

**Figure 4:**
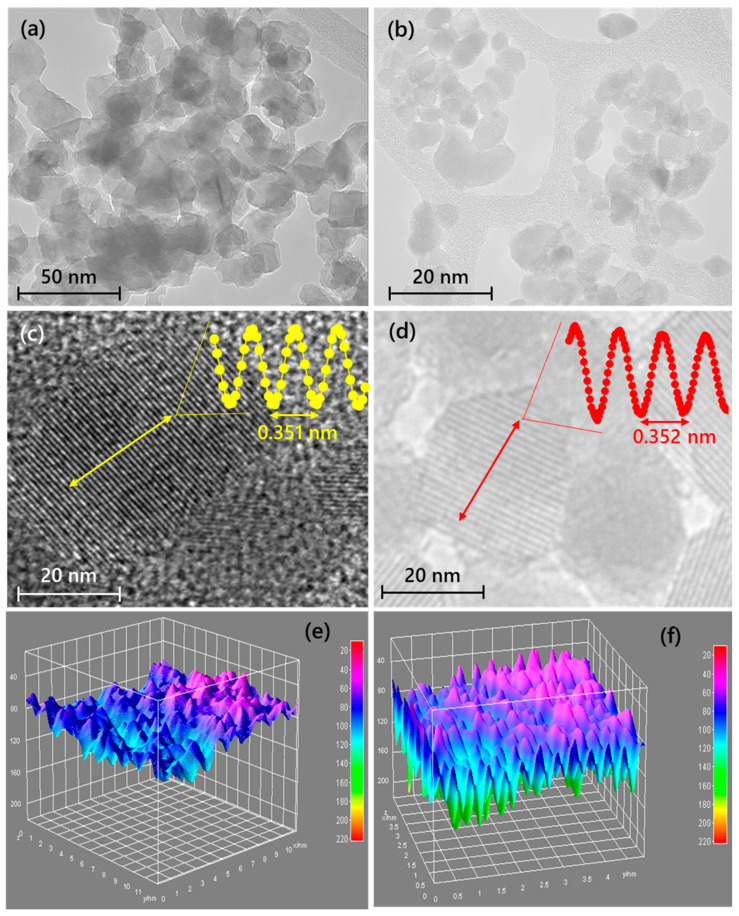
TEM images of (**a**) CB-TiO_2_, (**b**) CNB-TiO_2_, HRTEM images of (**c**) CB-TiO_2_, (**d**) CNB-TiO_2_, and 3D view surface plots of (**e**) CB-TiO_2_, and (**f**) CNB-TiO_2_. Inset of (**c**,**d**) are the corresponding line profiles CB-TiO_2_ and CNB-TiO_2_, respectively.

High-resolution transmission electron microscopy (HRTEM) analysis was performed to get complementary information about the structural properties and morphology of the CB-TiO_2_ and CNB-TiO_2_. Figure 4a,b presents the TEM images of the CB-TiO_2_ and CNB-TiO_2_, respectively. The TEM micrographs revealed the spindle-like morphology of both the samples with a small fraction of spherical-shaped particles. The d-spacing obtained from XRD analysis was further verified from HRTEM micrographs. The HRTEM observation confirmed that the interplanar distance between the adjacent lattice fringes was 0.351 and 0.352 nm for CB-TiO_2_ and CNB-TiO_2_, respectively. The HRTME analysis also confirmed the anatase phase of the CB-TiO_2_ and CNB-TiO_2_, indicated by the d-spacing of 0.351 and 0.352 nm for (101) anatase planes which is consistent with XRD results. The line profiles constructed from the selected area highlighted in yellow and red colored straight lines shown in the inset of Figure 4c,d suggest no structural deviation from the anatase crystalline structure. Furthermore, the 3D surface plots taken from the inner region of the samples shown in Figure 4e,f suggest the properly ordered atomically flat surfaces of both the samples, indicating that the long-range atomic arrangement was intact even after C and C-N doping of the black TiO_2_. However, the surface of the CNB-TiO_2_ shows a more proper arrangement of the atoms compared to CB-TiO_2_.

**Figure 5:**
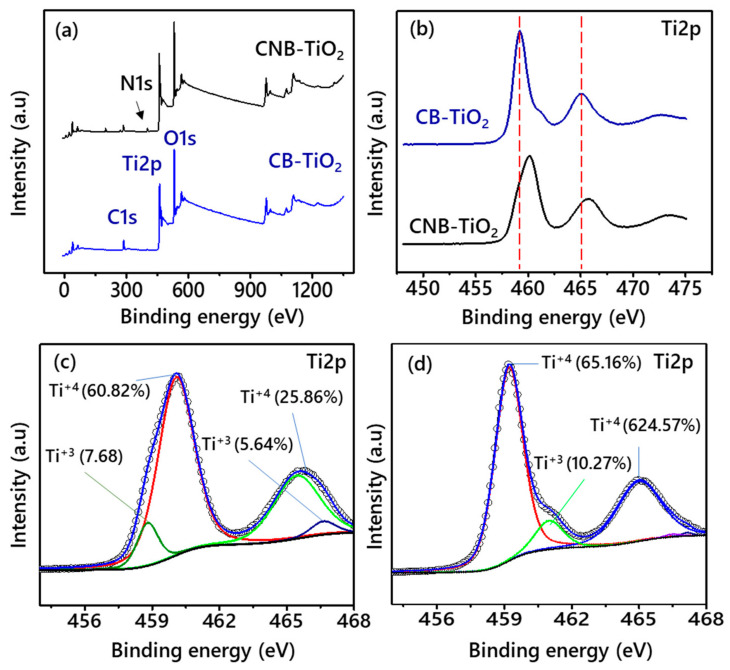
(**a**) XPS survey spectra and Ti2p XPS spectra (**b**) without deconvolution, and high resolution deconvoluted spectra of (**c**) CB-TiO_2_, and (**d**) CNB-TiO_2_.

XPS analysis was carried out to obtain information about the overall chemical environment and surface chemical composition of the CB-TiO_2_ and CNB-TiO_2_. Figure 5a shows a comparison of the XPS survey spectra of both samples. The survey spectra confirmed the presence of C in the CB-TiO_2_ and C and N in the CNB-TiO_2_. The binding energy (BE) values for Ti, O, C, and N peaks are listed in Table 2.

Figure 5b depicts a comparison of the Ti2p XSP spectra of the CB-TiO_2_ and CNB-TiO_2_. It is evident that the Ti2p peaks of the CBN-TiO_2_ shifted to a higher BE compared to CB-TiO_2_ nanomaterial. This suggests the different nature of the chemical bonding and coordination environment of the CB-TiO_2_ and CNB-TiO_2_. The Ti2p spectra of the samples were deconvoluted using Gaussian line fitting with the smart background. The Ti2p peaks shown in Figure 5c arising from orbital spin doublet of CB-TiO_2_ were positioned at a BE of 459.18 and 464.68 eV for Ti2p_3/2_ and Ti2p_1/2_, respectively, which are located at a relative higher BE compared to pure anatase phase TiO_2_ with Ti^4+^‒O bonds reported previously [35,36].

The Ti2p_3/2_ and Ti2p_1/2_ peaks of CNB-TiO_2_ shifted to a higher BE compared to CB-TiO_2_, and the peaks are now centered on 460.08 and 466.63 eV, as shown in Figure 5d. The positive shift in peak positions might be due to the creation of Ti^3+^ species, which results in an increase of the Ti–O bond length and thus expanding the BE of Ti2p of CNB-TiO_2_ [37,38]. Furthermore, the introduction of N into the TiO_2_ lattice results in a simultaneous increase in Ti2p BE, which is associated with a difference in the coordination environment of Ti and O atoms. The additional low-intensity peak was detected in Ti2p XPS spectra of the CNB-TiO_2_ at a BE of 460.98 eV (Ti2p_1/2_), which was assigned to the Ti^3+^ defects [39]. Similar shoulder peaks with a lower intensity were also observed in the Ti2p XPS spectra of CB-TiO_2_, as displayed in Figure 5d, which were assigned to the Ti^3+^ defects. The exact concentration of Ti^3+^ and Ti^4+^ shown in Figure 5c,d was determined from their respective peak areas, considering the sensitivity factors of the elements. The Ti^3+^ defects concentration was higher in CB-TiO_2_ than the CBN-TiO_2_.

**Figure 6:**
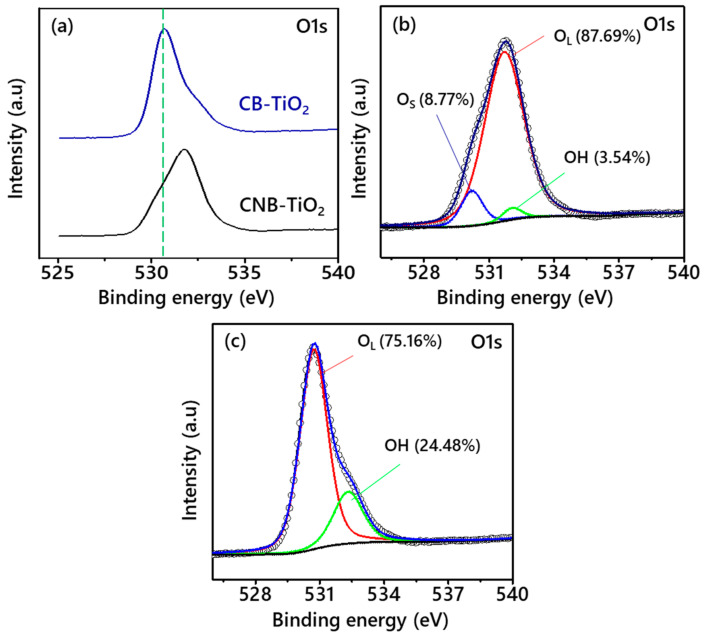
O1s XPS spectra (**a**) without deconvolution, and high resolution deconvoluted spectra of (**b**) CB-TiO_2_, and (**c**) CNB-TiO_2_.

Figure 6a illustrates a comparison of the O1s spectra of CB-TiO_2_ and CNB-TiO_2_. The O1s CNB-TiO_2_ peaks show a positive shift towards higher BE compared to CB-TiO_2_. Moreover, CNB-TiO_2_ exhibits a broader O1s peak. Figure 6b depicts the deconvoluted O1s peaks of CB-TiO_2_.

The O1s spectra present three main peaks located at a BE of 530.18, 531.68, and 532.06 eV, which are attributed to the lattice oxygen in TiO_2_, suboxide oxygen and OH groups, respectively. On the other hand, the O1s spectra of CNB-TiO_2_ in Figure 6c present two peaks at 530.68 and 532.28 eV, which can be ascribed to lattice oxygen (O_L_) in TiO_2_ and OH groups [39]. The amount of lattice oxygen in CB-TiO_2_ is 87.69% higher than that of CNB-TiO_2_ (75.16%). The amount of OH group is higher (24.48%) in CNB-TiO_2_ compared to only 3.54% in CB-TiO_2_.

**Figure 7:**
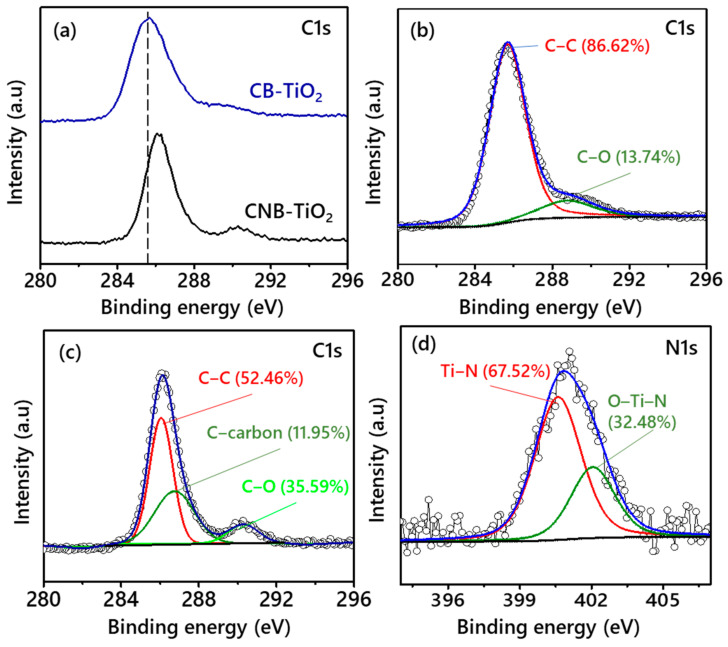
(**a**) Comparison of C1s spectra CB-TiO_2_ and CNB-TiO_2_, (**b**) deconvoluted C1s spectra of CB-TiO_2_, (**c**) deconvoluted C1s spectra of CNB-TiO_2_, and (**d**) deconvoluted N1s spectra of CNB-TiO_2_.

Figure 7a shows a comparison of the C1s spectra of the CB-TiO_2_ and CNB-TiO_2_. It can be observed that the C1s peak of CNB-TiO_2_ is shifted to a higher BE compared to CB-TiO_2_. The positive shift of C1s peak is consistent with the Ti2p and O1s peaks, which also show a shift towards higher BE. These results suggest that the chemical environment of the CNB-TiO_2_ is changed with the incorporation of N into its matrix. To get some more insight into the chemical environment of the samples, their C1s spectra was deconvoluted. Figure 7b depicts the high resolution deconvoluted C1s spectra of the CB-TiO_2_. The peaks were located at a BE of 285.68 and 288.86 eV, which represent residual carbon (C-C) with sp^2^ hybridization and C atoms substituting Ti in the TiO_2_ lattice (C-O), respectively [40]. An additional peak was observed at a BE of 286.76 eV in Figure 7c for CNB-TiO_2_, which can be attributed to coke carbon [41]. The N1s spectra of CNB-TiO_2_ was deconvoluted into two peaks centered on 400.60 and 402.06 eV, which corresponds to the substituted atomic N in the TiO_2_ lattice (Ti–N), N anions at interstitial site represented as O–Ti–N linkage, respectively. The substitutional N plays a crucial role in modifying the electrical conductivity (EC) and narrowing the bandgap of TiO_2_ as evident from Figure 3c, whereas the interstitial N can only introduce some isolated localized N2p states in the bandgap [42]. Shown in Figure 7d, the substituted N is higher than the interstitial one, which is expected to enhance the electrical conductivity of the CNB-TiO_2_.

### 3.2. Comparative Photocatalytic Performance of CB-TiO_2_ and CNB-TiO_2_ Nanomaterials

**Figure 8:**
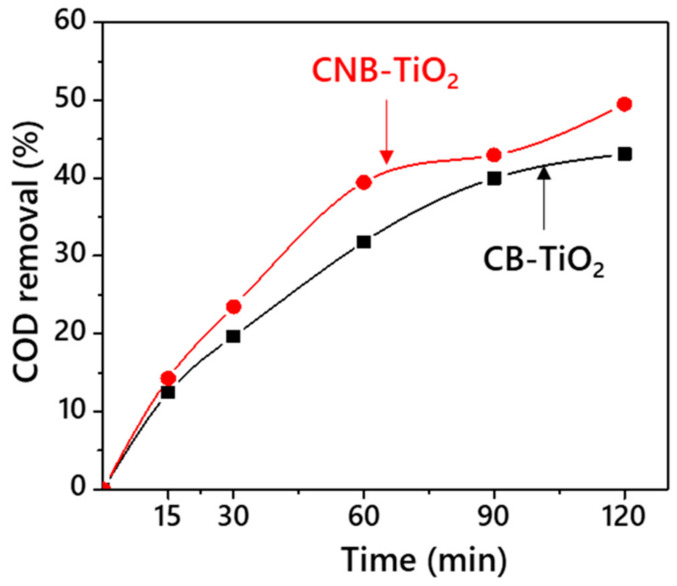
Comparative performance of CB-TiO_2_ and CNB-TiO_2_ for COD removal from the TPOME.

The photocatalytic performance of the CB-TiO_2_ and CNB-TiO_2_ was evaluated for the COD removal from TPOME under visible light irradiation to determine the best performing photocatalyst which will then be used in the sonophotocatalytic reaction. For this purpose, a fixed amount (0.4 g/L) of each sample was suspended in TPOME solution and stirred in the dark to achieve adsorption-desorption equilibrium. Afterwards, it was irradiated with visible light. A comparison of the percentage of COD removal by CB-TiO_2_ and CNB-TiO_2_ is shown in Figure 8. A noticeable improvement in COD removal can be witnessed for CNB-TiO_2_ compared to CB-TiO_2_. The CB-TiO_2_ was able remove 45.72% of COD from initial 800 mg/L within 120 min of visible light irradiation, whereas the CNB-TiO_2_ removed 51.48% of COD from the TPOME within the same time. The enhanced performance of the CNB-TiO_2_ can be attributed to its lower bandgap and improved visible light absorption. The appropriate amount of Ti^3+^ defects (10.27%) could have created inter-band energy levels in CNB-TiO_2_, which lead to the efficient visible light absorption to photoexcite electrons from the Ti^3+^ centers to the CB, and therefore enhance the visible light photocatalytic performance [43]. In case of CB-TiO_2_, the lower performance in terms of COD removal may be due to the following reasons: firstly, the CB-TiO_2_ lower surface area (239.99 m^2^/g), as shown in Table 2, leading to the lowest number of active sites thus reducing its ability to contact reactants; and secondly, it possesses a higher content of Ti^3+^ species, resulting in producing additional localized states below the CB which might be unfavorable to achieve better photocatalytic performance [44,45]. Because too high concentration of Ti^3+^ species can act as charge carrier recombination centers, this results in low carrier mobility [46]. Low carrier mobility implies that a small number of electrons are disassociated from the surface of the photocatalyst to take part in photoreaction. Kato et al. [47] reported that the excess concentration of Ti^3+^ species in gray TiO_2_ served as charge carrier recombination sites, leading to lower photocatalytic performance. The comparative photocatalytic performance confirmed the superior performance of CNB-TiO_2_; therefore, it is further employed in sonophotocatalytic reactions.

### 3.3. Oxidation of TPOME Using Various AOPs

The oxidation of TPOME was investigated using various AOPs, including sonolysis, photolysis, catalysis, sonophotolysis, sonocatalysis, photocatalysis, and sonophotocatalysis. The oxidation of TPOME was monitored by the removal of COD. The COD removal efficiency of each single and combined process is summarized in Table 3. A single AOP, such as photolysis, sonolysis, and catalysis could achieve less than 10% COD removal efficiency from TPOME. The low COD removal efficiency by only US is understandable, given the low US frequency adopted in the current study (30 kHz), which produces a small number of cavitation bubbles and consequently leads to low performance [23]. The COD removal efficiency from TPOME enhanced when the two processes were combined. For example, when US and visible light were combined (sonophotolysis), the COD removal efficiency increased to 17.84%. Combining photolysis and US resulted in an incremental increase in COD removal efficiency and the efficiency rose to 20.34%, which could be attributed to the synergistic effect of light and the US waves [48].

Combining US and TiO_2_ (sonocatalysis) showed higher COD removal efficiency (20.34%) compared to sonophotolysis (17.84%) after 180 min of reaction time. The US and TiO_2_ work in synergism, where the pores that present on the surface of TiO_2_ function as cavitation nuclei to promote the formation of cavitation bubbles, followed by the growth of cavitation bubbles (expansion), and the cavitation bubbles finally explode, adiabatically enhancing the removal of organics [49,50].

The COD removal efficiency from TPOME was drastically enhanced to 51.48% under visible light in the presence of CNB-TiO_2_ (photocatalysis). As expected, when CNB-TiO_2_ was irradiated with visible light, it vacated electrons from the VB to the CB, leaving behind a hole in the VB, as shown in Equation (6). The holes react with hydroxide ions (OH^−^) to produce ^•^OH radicals (Equation (7)). The ^•^OH radicals are the predominant reactive species taking part in photoreactions, leading to the degradation of organic molecules in TPOME. The anatase phase CNB-TiO_2_ used in the current study is reportedly producing ^•^OH radicals faster as compared to other photocatalysts [51], and thus resulting in the higher COD removal efficiency from TPOME.

(6) $${\mathrm{TiO}}_{2}+\mathrm{hv}\to e_{\mathrm{CB}}^{-}+{\;h}_{\mathrm{VB}}^{+}$$

(7) $$h_{\mathrm{VB}}^{+}+{\mathrm{OH}}^{-}\to {}^{•}\mathrm{OH}$$

On the other hand, combining US and photocatalysis (sonophotocatalysis) achieved 64.17% COD removal efficiency from TPOME, which is the highest among all the combinations of processes tested in the present study. The synergistic response of combining US + CNB-TiO_2_ + Visible (sonophotocatalysis) could be explained as follows: the CNB-TiO_2_ with a relatively higher surface area (347.26 m^2^/g) offers more chances for the adsorption of the organic on its surface; the visible light will excite the electron from the VB of CNB-TiO_2_ and produce holes, which may react with water to produce oxidative ^•^OH radicals which attack the organics molecules; and the US cavitation will promote the generation of additional ^•^OH radicals [52], which will eventually enhance the removal of COD from TPOME. Furthermore, US helps continuously regenerate the TiO_2_ photocatalyst surface and expose active sites for organic adsorption and consequent degradation. When the CNB-TiO_2_ is irradiated with visible light in the presence of US, organics molecules are oxidized by both the excited electron in the CB of CNBTiO_2_ and the US splitting of H_2_O molecules, as explained in the following equations (Equations (8)–(12)) [53,54]:

(8) $$\mathrm{CNB}‒{\mathrm{TiO}}_{2}\overset{\mathrm{hv}}{\to }\;\mathrm{CNB}‒{\mathrm{TiO}}_{2}\left(e^{-}\right)+{\;\mathrm{TiO}}_{2}\left(h^{+}\right)$$

(9) $$h^{+}+{\;\mathrm{OH}}_{\mathrm{ads}}^{-}\;\to {}^{•}\mathrm{OH}$$

(10) $$e^{-}+{\;O}_{2}\to {\;O}_{2}^{•-}\to {}^{•}\mathrm{OH}$$

(11) $$H_{2}O\;\overset{))))}{\to }{}^{•}H\;+\;{}^{•}\mathrm{OH}$$

(12) $${}^{•}\mathrm{OH}\;+\;{}^{•}\mathrm{OH}\to H_{2}O_{2}$$

where hv is the photon required to excite $\left(e^{-}\right)$ from the VB of CNB-TiO_2_ to the CB leaving a positive hole behind in the VB represented as $\left(h^{+}\right)$, and ^•^OH^–^ is a hydroxide ion present on the surface.

The production of ^•^OH radicals by CBN-TiO_2_ is explained in Equations (7)–(9). The cavitation bubbles were formed when US was introduced into TPOME in the presence of CNB-TiO_2_. As shown in Equation (12), the heat from the cavitation explosion split water molecules into extremely reactive hydroxyl radicals. The radicals penetrate into the solution and degrade the organic contents present in TPOME. The US helps in increasing soluble organics present in TPOME, which are more amenable to photocatalytic oxidation [55]. Thus, the efficiency of photocatalysis coupled with US is higher compared to other processes investigated in the present study, and is in good agreement with previous studies on the sonophotocatalytic treatment of olive oil mill wastewater, which has similar characteristics to TPOME [56]. The results suggest that visible light-mediated photocatalysis based on CBN-TiO_2_ coupled with US cavitation is a promising approach for TPOME’s remediation. Thus, sonophotocatalysis was further investigated for COD removal from TPOME considering the effect of various operating parameters, including CBN-TiO_2_ loading, initial COD concentration, H_2_O_2_ dosage, US power, and US frequency, which are discussed in the following sections in detail.

#### 3.3.1. Effect of CNB-TiO_2_ Loading

**Figure 9:**
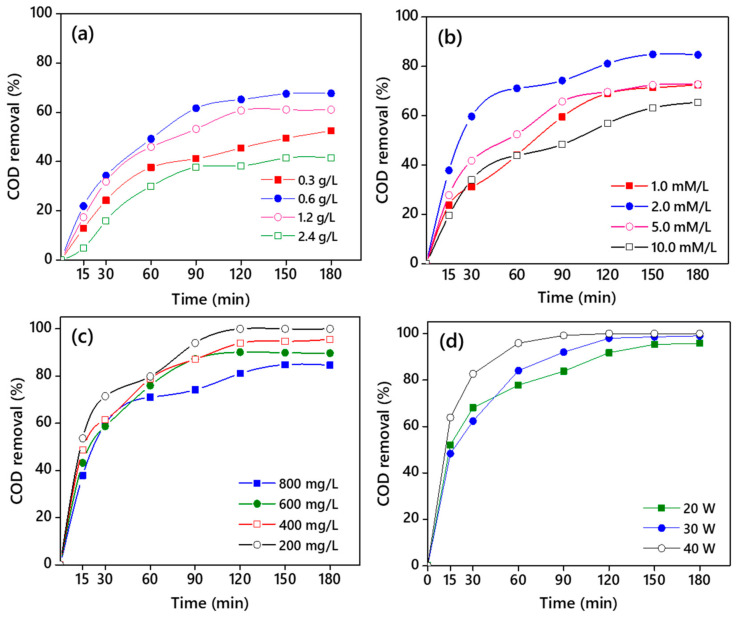
(**a**) COD removal efficiency from TPOME via sonophotocatalysis (**a**) effect of CNB-TiO_2_ loading (**b**), effect of H_2_O_2_ dosage (**c**), effect of COD concentration, and (**d**) effect of ultrasound power.

CBN-TiO_2_ loading showed a significant effect on COD removal from TPOME. Four different CNB-TiO_2_ loadings were studied, and the results are displayed in Figure 9a. At all the loading levels, the maximum COD removal was achieved within the initial 90 min of the reaction after which the COD removal efficiency almost flattened. This can be attributed to a large number of active sites on the CNB-TiO_2_ surface available for organic adsorption, and subsequently the production of more ^•^OH radicals available at the initial phase of the sonophotocatalytic reaction. The lower COD removal at a later stage during the reaction can be due to the complete coverage of the CNB-TiO_2_ surface by the organics, blocking the active sites [13]. The COD removal efficiency increased to 67.69% with increasing the CNB-TiO_2_ loading to 0.6 g/L compared to 46.50% at 0.3 g/L. At low loading (0.3 g/L), the organic molecules did not have sufficient active sites available on the surface of CNB-TiO_2_ particles, which consequently resulted in a decrease in COD removal efficiency from TPOME. However, the COD removal efficiency decreased to 41.51% at a CNB-TiO_2_ loading of 2.4 g/L. Similar results of an increase in COD removal with increasing TiO_2_ loading up to a certain level (1.0 g/L) have been previously reported by Cheng et al. [57]. Beyond 1.0 g/L, the COD removal efficiency markedly decreased at 1.5 and 2.0 g/L loadings. There can be three possible reasons for the lower COD removal at higher CNB-TiO_2_ loading: (i) the excessive CNB-TiO_2_ loading leads to the agglomeration of the particles, thus reducing the available surface area for organics adsorption; (ii) a high loading diminishes the number of photons to strike the surface of photocatalyst, which retards the generation of oxidative and reductive species; and (iii) high loading causes turbidity of the solution and increases the light attenuation by nanoparticles [58]. At a high loading of CNB-TiO_2_, beyond a loading of 0.6 g/L, the COD removal efficiency from TPOME decreased even in the presence of US. The US may have helped increase the light scattering effect, and consequently suppressed the passage of light through the TPOME suspension. These results are consistent with those obtained by [56], where the maximum COD removal (~30%) from olive oil mill wastewater, which has similar characteristics to TPOME, was obtained at an optimal TiO_2_ loading of 0.75 g/L. It is important to note that the final COD concentration after treatment was ~260 mg/L, which is still above the maximum permissible limit (50 mg/L) for COD in TPOME. This could be due to the higher initial concentration of COD (800 mg/L) in TPOME compared to previous studies (150–170 mg/L) because high initial substrate concentration decreases the performance of the treatment system. To increase the COD removal efficiency and produce an effluent with a COD concentration within the permissible limit, two types of strategies were adopted: (i) H_2_O_2_ was added to the system; and (ii) the initial COD concentration was adjusted through dilution. The effect of H_2_O_2_ dosage on sonophotocatalytic removal of COD from TPOME is explained in the following section.

#### 3.3.2. Effect of Hydrogen Peroxide (H_2_O_2_) Dosage

The effect of different H_2_O_2_ dosages on COD removal efficiency from TPOME was investigated, as illustrated in Figure 9b. The sonophotocatalytic removal of COD increased from 67.69% to 75.48% with the addition of 1.0 mM H_2_O_2_. The COD removal efficiency further increased to 84.63% when the H_2_O_2_ concentration was increased to 2.0 mM. The incremental increase in COD removal efficiency with the addition of H_2_O_2_ suggests the synergistic effect of CNB-TiO_2_ photocatalyst and H_2_O_2_. H_2_O_2_ lessens the recombination of electron-hole pair by trapping the negative charged electron in the CB of CNB-TiO_2_, and produces additional ^•^OH free radicals which amplify the ^•^OH free radicals produced by CNB-TiO_2_. Thus, more reactive ^•^OH radicals are available to degrade organic molecules in TPOME [59,60]. The positive effect of H_2_O_2_ during sonophotolysis reaction on COD removal efficiency from various industrial effluent has been previously reported by different authors [61,62,63].

The mechanism of action of H_2_O_2_ and TiO_2_ photocatalyst on degradation during the sonophotocatalytic process can be described by the following Equations (Equations (13)–(16)) [60]:

(13) $${\mathrm{TiO}}_{2}\left(e^{-}\right)+H_{2}O_{2}\;\overset{))))))}{\to }{\;\mathrm{TiO}}_{2}+{\;\mathrm{OH}}^{-}+{}^{•}\mathrm{OH}$$

(14) $$H_{2}O_{2}+{\;e}^{-}\left(\mathrm{CB}\right)\;\to {}^{•}\mathrm{OH}\;+\;{\mathrm{OH}}^{-}{\;}^{\;}$$

(15) $$H_{2}O_{2}+\mathrm{hv}\;\to 2{}^{•}\mathrm{OH}$$

(16) $$H_{2}O_{2}+{\;O}_{2}\;\to {}^{•}\mathrm{OH}\;+\;{\mathrm{OH}}^{-}+O_{2}\;$$

As can be observed in Figure 9b, the COD removal efficiency decreases with increasing H_2_O_2_ dosage beyond 2.0 mM. The COD removal efficiency from TPOME decreased to 71.36% when the H_2_O_2_ dosage was increased to 10.0 mM. This is because the high concentration of H_2_O_2_ induces a scavenging effect, and reduces the removal of organic compounds in TPOME, as shown in Equations (17) and (18).

(17) $$H_{2}O_{2}\;+\;{}^{•}\mathrm{OH}\to {\;\mathrm{OH}}_{2}^{•}+{\;H}_{2}O$$

(18) $${\mathrm{OH}}_{2}^{•}\;+\;{}^{•}\mathrm{OH}\to {\;H}_{2}O+{\;O}_{2}$$

A total of 677.07 mg/L of COD was removed from the initial 800 mg/L in TPOME in 180 min of sonophotocatalytic treatment in the presence of 2.0 mM H_2_O_2_ as shown in Figure 9b. Despite this, the final COD concentration (122.93 mg/L) in TPOME was still above the maximum permissible limit set by the DOE Malaysia. To overcome this problem and further increase the COD removal efficiency, the effect of dilution or initial COD concentration was investigated.

#### 3.3.3. Effect of Initial COD Concentration

The effect of the initial COD concentration was investigated on sonophotocatalytic removal of COD from TPOME. Four different initial concentrations of COD at pH 7.6 were studied at the optimal TiO_2_ loading of 0.6 g/L and 2.0 mM of H_2_O_2_ dosage. The initial concentration in TPOME was adjusted by dilution at 0, 75, 50, and 25% dilution to obtained 800, 600, 400, and 200 mg/L COD, respectively. The COD removal efficiency from TPOME by the sonophotocatalytic process at different initial COD concentrations is presented in Figure 9c. The COD removal efficiency was higher at a low initial concentration compared to a higher initial concentration. For instance, the COD removal efficiency was 84.63% at 800 mg/L initial concentration of COD in TPOME. At 400 mg/L, the COD removal efficiency was 95.53% and the final effluent COD (18.86 mg/L) was lower than the permissible limit of 50 mg/L.

At 200 mg/L, 100% of COD was removed from TOPME in 120 min of reaction, suggesting that the dilution not only enhances the COD removal efficiency but also reduces the reaction time to achieve maximum COD removal efficiency. A higher substrate concentration reduces the photocatalytic degradation efficiency, due to the following three reasons: (1) higher initial concentration reduces the ability of light to reach the surface of the CNB-TiO_2_; (2) it diminishes photonic efficiency, which results in saturation of the CNB-TiO_2_ and its deactivation; (3) it can lead to a large number of intermediate formations, which may adsorb on the surface of the CNB-TiO_2_, making it dysfunctional [61,64]. The results of the present study are in agreement with previous work, where the COD removal from TPOME is reportedly decreased from 98.2 to 52.5%, with a rise in the initial concentration of COD from 6 to 28 mg/L, respectively [12]. The COD concentration in TPOME after the treatment at 400 mg/L and 200 mg/L was well below the permissible limit represented. However, the COD removal was extremely fast at 200 mg/L compared to 400 mg/L, indicated by their reaction time of 60 min and 120 min, respectively.

#### 3.3.4. Effect of Ultrasound Power

The power of ultrasound waves received by the reaction media is an important factor in the sonophotocatalytic process because the estimated energy consumption by the sonophotocatalytic process strongly depends on US power [65]. The effect of US power on COD removal efficiency from TPOME using sonophotocatalysis was evaluated, and the results are presented in Figure 9d. The US power was adjusted at three different amplitudes, such as 35, 50, and 70% to get a US power of 20, 30, and 40 W. The reaction was carried out at 0.6 g/L of TiO_2_ loading, 2.0 mM of H_2_O_2_ dosage, and 200 mg/L of COD initial concentration. The results suggest that the US power has a significant effect on COD removal efficiency, and 100% of COD from TPOME was removed by the three different US power with different reaction times. However, the 40 W US power was extremely efficient, removing >99% of the COD from TPOME within 60 min of reaction time, and the final effluent COD was well below the maximum permissible limit (50 mg/L) set by the Malaysian Department of Environment. It takes 120 and 150 min for a US power of 20 and 30 W, respectively, to remove >99% of the COD from TPOME to reduce the COD levels to the allowable limits. It is generally accepted that increasing US power increases the COD removal efficiency because of the increase in the number of US-produced bubbles, which subsequently increases the number of ^•^OH radicals available to attack the target pollutant [66]. Considering the energy consumption by US, the 20 W US power is the most appropriate, as it will reduce the energy consumption by using low power and less electrical energy. However, the high energy consumption by a US power of 40 W can be compensated by the lower reaction time of 60 min compared to 120 and 150 min by US power of 20 and 30 W, respectively. Certainly, there is a tradeoff between higher efficiency and the higher electrical power used by the US. Furthermore, the use of costly H_2_O_2_ can also be compensated by the low reaction time of 60 min at 40 W of US power compared to 120 and 150 min.

### 3.4. Kinetic Study Synergy Index

**Figure 10:**
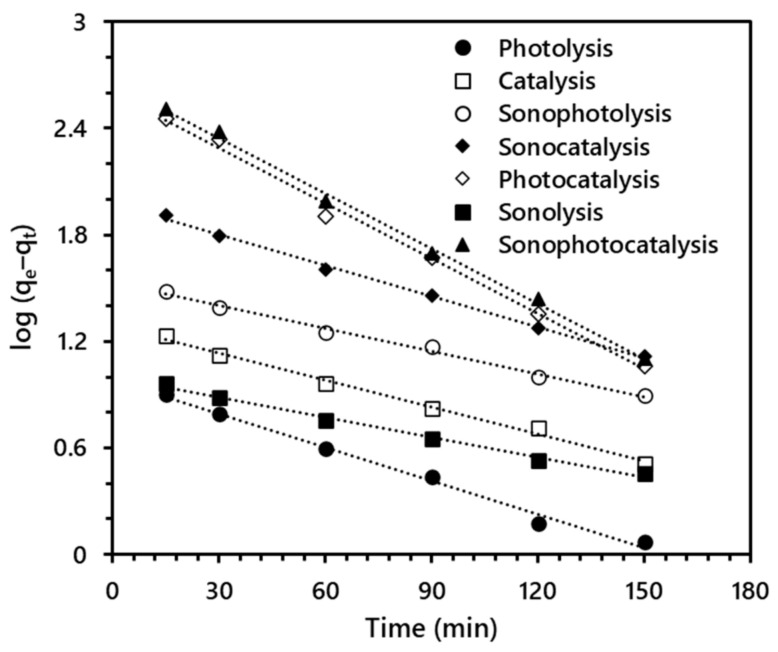
Pseudo-first-order kinetic plot for COD removal from TPOME via different advanced oxidation processes.

Reaction kinetics studies help to investigate and understand the mechanism and performance of a system via the determination of the rate of reactions [67]. In the present study, the remediation efficiency of TPOME in terms of COD removal from TPOME by different AOPs was assessed using a pseudo-first-order kinetic model expression, as follows [68]:

(19) $$\log\;\left(q_{e}-q_{t}\right)=\log q_{e}-\frac{K_{1}t}{2.303}$$

where *K*_1_ is the COD removal rate constant in L/min, *q_e_*, and *q_t_* in mg/g represent the COD removed at equilibrium *e* and time *t*, respectively. The values for $\log\;\left(q_{e}-q_{t}\right)$ were plotted against the time (min), as shown in Figure 10, to obtained rate constant values from the slope and intercept. The rate constant and coefficient of determination (R^2^) values are presented in Table 3.

The R^2^ values ranging from 0.9913–0.9974 show the excellent fitting of the data into the pseudo-first-order kinetic model. The rate constant (*K*_1_) values for the combined processes are by far higher than the single process. The sonophotocatalytic process exhibited the highest rate constant of 2.395 × 10^–2^ for COD removal from TPOME. Based on the rate constant values reported in Table 3, the synergy index of 0.912 was calculated for COD removal from TPOME in the sonophotocatalytic process. The synergy index value obtained in the present study is lower than the previously reported value (1.35) for COD removal, using nanosized TiO_2_ and US frequency of 20 kHz under UV irradiation [56]. The synergistic effect of the US coupled with photocatalysis has also been previously demonstrated on COD removal.

### 3.5. Energy Consumption

In sonophotocatalytic reactions, a major fraction of energy is required for ultrasound irradiation. In this study, the electrical energy (EE) consumption for the removal of a unit COD from TPOME by one order of magnitude was assessed using Equation (20) [65].

(20) $$\mathrm{EE}=\frac{P_{el}t\left(1000\right)}{V60\;log\left(\frac{C_{o}}{C_{t}}\right)}$$

where *P_el_* is the electrical power applied kW (5 kW for photocatalytic process, 0.02, 0.03, and 0.04 for sonication process); *t* denotes the sonophotocatalytic treatment time (h) required to treat a specific volume of TPOME (V) in L; 1000 is the factor used to change grams to kilograms; and *C_o_* and *C_t_* are the initial and concentration of COD at any time, respectively.

The calculated electrical energy required for the sonophotocatalytic process operated at different US powers, including 20, 30, and 40 W for COD removal from TPOME is displayed in Table 4. Based on the time required to achieve more than 90% of COD, the electrical energy calculated for the combination of processes (sonolysis and photocatalysis) was 1342.25 kWh/m^3^ for a US power of 20 W. The electrical energy needed for 40 W US power was the lowest (177.59 kWh/m^3^) because using 40 W power reduced the reaction time by almost 200% compared to 20 W. In the present work, the electrical energy per order used by the sonophotocatalytic process was far lower than the previously reported 3217 kWh/m^3^ for COD removal from TPOME [12]. This could be because of the extended treatment time in previous works compared to the present work. The results suggest that the sonophotocatalytic process not only reduces the COD concentration to the stipulated standard discharge limit but also reduces the treatment time and energy consumption. It can be concluded that the synergetic effect of photocatalysis based on CNB-TiO_2_ and US improve the remediation efficiency of TPOME, lower the energy requirement, and reduce the treatment time drastically.

### 3.6. Recyclability of the CBN-TiO_2_ Nanomaterial

**Figure 11:**
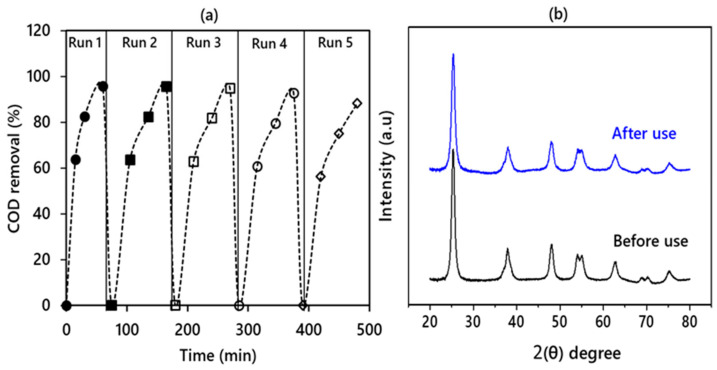
(**a**) Recyclability of the CNB-TiO_2_ after five repeated runs and (**b**) XRD patterns of black CNB-TiO_2_ before and after sonophotocatalytic reaction.

The recovery of TiO_2_ nanoparticles is of great interest, since the release of effluent after the treatment containing nanoparticles can cause environmental and human health impacts. The CBN-TiO_2_ nanomaterial was retrieved after the sonophotocatalytic treatment. The recovered CBN-TiO_2_ nanomaterial was used for five consecutive runs for the removal of COD from TPOME and the results are shown in Figure 11a. Since the TiO_2_ particles are generally chemically and thermally stable, therefore, a decrease of not more than 5% was observed in its activity after five consecutive runs. This can be due to the positive effect of US, as it continuously regenerates the surface of the photocatalyst and exposes more active sites on its surface for enhanced organic adsorption and subsequent degradation.

To further confirm the stability of CBN-TiO_2_ nanomaterial, the XRD patterns of the sample were compared before and after the use in five consecutive runs. Figure 11b depicts the XRD patterns of the CBN-TiO_2_ nanomaterial before and after the use. The XRD patterns reveal only a slight decrease in intensity and broadening of the anatase peak (101) around 25.35 °C (2θ) without any crystal structure distortion. Thus, this study corroborated that the CBN-TiO_2_ nanomaterial could be recycled and employed for remediation.

### 3.7. Sonophotocatalytic Degradation of TPOME under Optimal Conditions

The sonophotocatalytic process for the COD removal from TPOME was operated under the optimized conditions of US frequency: 30 kHz, CNB-TiO_2_ loading: 0.6 g/L, H_2_O_2_ dosage: 2.0 mM, initial COD concentration: 200 mg/L, US power: 40 W, and treatment time of 60 min. Four experimental runs were operated under the optimized experimental conditions to validate the optimized conditions and the results are reported as averages. More than 99% of the COD was removed from TPOME within 1 h of sonophotocatalytic reaction. Comparing the results obtained in the present study with those reported in the literature reveals higher COD removal efficiency from TPOME using the sonophotocatalytic process. In previous studies, Ng et al. [67] reported 55% of COD removal efficiency using ZnO photocatalyst under UV irradiation and at the optimum conditions of 60 mL/min oxygen flow rate, 1.26 g/L of ZnO concentration, and 220 ppm of initial COD concentration. Taha et al. [3] reported a 75% COD removal efficiency from treated POME employing Fenton oxidation process. Cheng et al. [68] reported 26.77% of COD removal using photocatalytic process based on silver doped titanium dioxide (Ag/TiO_2_) under visible light irradiation. Remarkably, >99% COD removal efficiency in the present study is comparatively higher than the previously reported efficiencies, which is an indication that the fabrication of visible light active CNB-TiO_2_ is crucial for enhancing visible light driven sonophotocatalytic remediation of TPOME. It also signifies that the experiments were carried out precisely at a laboratory scale and highlight the importance of integrating two processes; photocatalysis and ultrasound irradiation is an effective approach to remove COD from TPOME to achieve the standard limit.

## 4. Conclusions

In the present study, carbon and carbon-nitrogen doped TiO_2_ were produced, and their photocatalytic performance was evaluated for remediation of treated palm oil mill effluent. The characterization results demonstrated excellent optical properties of the carbon-nitrogen doped TiO_2_ nanomaterials. Carbon-nitrogen doped TiO_2_ nanomaterials also showed enhanced COD removal from TPOME compared to carbon doped TiO_2_. An investigation on the removal of COD from treated palm oil mill effluent was also conducted using various advanced oxidation processes and their combination. The results demonstrated that the combination of photocatalysis and ultrasonication assisted by H_2_O_2_ under the visible light was more efficient for COD removal from TPOME compared to individual processes. The maximum COD removal efficiency of the combined process (sonophotocatalysis + H_2_O_2_) was 84.63% compared to only 4.75, 51.48%, and 64.17% by sonolysis, photocatalysis, and sonophotocatalysis, respectively. The enhanced COD removal by the combined processes was attributed to their synergistic effect, indicated by the increasing synergy index where the US augmented the ^•^OH radicals produced by the micron-sized TiO_2_ particles and H_2_O_2_ further increased the number of ^•^OH radicals and prevented the electron-hole pair recombination in TiO_2_. More than 90% of the COD removal was achieved within 60 min of sonophotocatalytic reaction at an initial COD concentration of 200 mg/L, and the final effluent COD was below the maximum permissible limit of 50 mg/L. The ultrasonication (US) power showed a significant effect on COD removal and the COD removal was the highest at 40 W US power. The energy consumption by the sonophotocatalytic process was 177.59 kWh/m^3^ by one order of magnitude, which is lower than 20 and 30 W because of the lower reaction time of 60 min. Most importantly, carbon-nitrogen co-doped TiO_2_ showed excellent stability indicated by a fractional loss (less than 5%) of the activity. Overall, the sonophotocatalytic treatment process investigated in this study exhibited a promising performance, and it can represent a viable approach for COD removal from TPOME, in terms of electrical energy consumption and environmental impact compared to conventional treatment requiring high energy and chemical inputs.

## Figures and Tables

**Table 1:** Properties of the CB-TiO_2_ and CNB-TiO_2_ nanomaterials.

Properties	CB-TiO_2_	CNB-TiO_2_
2θ anatase (101)	25.3704	25.2754
d-spacing (nm)	3.51073	3.52371
FWHM	0.3883	0.7675
Crystallite size (nm)	22.18	11.09
Bandgap (eV)	2.63	2.06
Absorption edge (nm)	500	630
Surface area (m^2^/g)	239.99	347.26
Pore size (nm)	3.7321	3.20380
Pore volume (cm^3^/g)	0.192781	0.254811

**Table 2:** XPS fitting parameters for CB-TiO_2_ and CNB-TiO_2_ nanomaterials.

Fitting Parameters	Binding Energy (eV)	
	CB-TiO_2_	CNB-TiO_2_
Ti2p_3/2_ (Ti^4+^)	459.18	460.08
Ti2p_1/2_ (Ti^3+^)	464.68	466.63
Ti2p_3/2_ (Ti^4+^)	458.78	-
Ti2p_1/2_ (Ti^3+^)	465.48	460.98
^1^ O_L_	530.18	530.68
^2^ O_s_	531.68	-
OH	532.06	532.28
C‒C	285.68	285.69
C‒O	288.86	288.76
Coke-C	-	286.76
O‒Ti‒N	-	400.60
Ti‒N	-	402.06

^1^ O_L_: lattice oxygen, ^2^ O_s_: suboxide oxygen.

**Table 3:** COD removal efficiency, kinetic rate constant, coefficient of determination, and synergy index of different advanced oxidation processes for COD removal from TPOME.

Advanced Oxidation Method	COD Removal Efficiency (%)	K_1_ (min^–1^)	R^2^	Synergy Index
Photolysis (Vis)	5.10 ± 0.48	1.451 × 10^−2^	0.9921	-
Sonolysis (US)	4.75 ± 0.42	8.751 × 10^−3^	0.9922	-
Catalysis (TiO_2_)	8.28 ± 0.24	1.175 × 10^−2^	0.9931	-
Sonocatalysis	20.34 ± 0.34	1.336 × 10^−2^	0.9974	0.6516
Sonophotolysis	17.84 ± 1.95	9.903 × 10^−3^	0.9913	0.4257
Photocatalysis	51.48 ± 0.61	2.394 × 10^−2^	0.9947	0.7326
Sonophotocatalysis	64.17 ± 0.76	2.395 × 10^−2^	0.9971	0.9118

**Table 4:** Comparison of the electrical energy consumption by different US power for >90% COD removal efficiency from TPOME at 0.6 g/L CNB-TiO_2_ loading, 2.0 mM H_2_O_2_, and 200 mg/L initial COD concentration.

US Power (W)	COD Removal (%)	Time (min)	Electrical Energy (kWh/m^3^)
20	>90	120	1342.25
30	>90	90	486.47
40	>90	60	177.59

## Data Availability

Not Applicable.
